# In vitro microleakage at the enamel and dentin margins of class II cavities of primary molars restored with a bulk‐fill and a conventional composite

**DOI:** 10.1002/cre2.729

**Published:** 2023-03-29

**Authors:** Shahram Mosharrafian, Niloofar Farahmand, Kiana Poorzandpoush, Zohre Sadat Hosseinipour, Mehdi Kahforushan

**Affiliations:** ^1^ Department of Pediatric Dentistry, School of Dentistry Tehran University of Medical Sciences Tehran Iran; ^2^ Department of Restorative Dentistry, School of Dentistry Kashan University of Medical Sciences Kashan Iran; ^3^ Department of Pediatric Dentistry, School of Dentistry Shahid Beheshti University of Medical Sciences Tehran Iran; ^4^ Department of Pediatric Dentistry, School of Dentistry AJA University of Medical Sciences Tehran Iran; ^5^ Private Practice Tehran Iran

**Keywords:** composite resins, dental enamel, dental leakage, dentin

## Abstract

**Objectives:**

This study assessed the enamel and dentin margin microleakage of class II cavities of primary molars restored with a bulk‐fill and a conventional composite.

**Materials and Methods:**

In this in vitro, experimental study, standard class II cavities were created in the proximal surfaces of 60 extracted primary molars. The teeth were randomly divided into two groups, and restored with SonicFill bulk‐fill and Filtek Z250 conventional composite along with Single Bond 2 adhesive. The teeth were coated with two layers of nail varnish to 1 mm around the restoration margins, and the apices were sealed with wax. The teeth underwent 1500 thermal cycles and incubated at 37°C for 24 h. They were then immersed in 1 M silver nitrate in the dark, rinsed with water, immersed in developing solution for 12 h, and exposed to fluorescent light. Next, they were mesiodistally sectioned, and digitally photographed under a stereomicroscope at ×10 magnification. The dye penetration depth was measured by a blind observer, and analyzed by the Mann–Whitney U test (*α* = .05).

**Results:**

No significant difference existed in microleakage between the two composite groups at the enamel (*p* = .76) or dentin (*p* = .16) margins. In both composite groups, microleakage at the dentin margins was significantly greater than that at the enamel margins (*p* = .000).

**Conclusion:**

Considering the absence of a significant difference in microleakage, SonicFill bulk‐fill composite can be used as an alternative to Filtek Z250 conventional composite for restoration of primary molars to benefit from its advantages such as simpler and faster application.

## 1 INTRODUCTION

By the advances in dental materials and clinical restoration techniques, direct composite resins are the most commonly used dental materials to meet the esthetic demands of patients in restoration of carious teeth, coronal fractures, dental erosions, and congenital defects (Kwon et al., 2012). Despite the optimal physical properties, polymerization shrinkage, and stress are the main drawbacks of conventional composite resins. Management of stress caused by polymerization shrinkage of dental composite resins is imperative to achieve optimal marginal integrity and guarantee the durability of restorations. Polymerization stress causes small cracks in the composite mass, and results in debonding of adhesive from the cavity walls, and subsequent gap formation and marginal microleakage, which results in postoperative tooth hypersensitivity. Marginal discoloration, low fracture resistance, caries recurrence, and tooth deformation are among other complications caused by polymerization stress (Radhika et al., 2010; Van der Vyver, 2010).

Microleakage refers to passage of bacteria, liquids, molecules, and ions through the cavity wall‐restoration interface, which is not clinically detectable (Vicente et al., 2009). Microleakage is an important factor that adversely affects the durability of restorations, and can cause tooth hypersensitivity, recurrent caries, and pulpal damage (Gong et al., 2019). To seal restoration margins, a uniform interface between the cavity walls and restoration is imperative (Gogna et al., 2011).

New chemical formulations of resins have been introduced to minimize microleakage. Also, it is important to facilitate and accelerate the restorative procedure by using restorative materials with fewer procedural steps. Bulk‐fill composite resins were introduced to the market to simply the restorative procedures and overcome the limitations of incremental application of conventional composite resins by the advances in their monomer, initiator, and filler technology. Compared with conventional resin composites, bulk‐fill composite resins have lower filler content and larger filler particles, as well as improved translucency (Ilie et al., 2013). Moreover, different chemical structures of monomers in bulk‐fill composites decrease their polymerization stress (Abbasi et al., 2018). However, bulk‐fill composite resins have a curing time comparable to that of conventional composites and are cured by the same curing units (Bucuta & Ilie, 2014). Bulk‐fill composite resins can be applied in 4‐mm thick increments and can preserve their optimal mechanical properties and degree of conversion in all thicknesses, which may be due to their decreased polymerization stress and high reactivity to light (Czasch & Ilie, 2013). Bulk‐fill composites have higher molecular‐weight monomers and novel initiator systems compared with conventional composites. Bulk‐fill composites are suitable for use in patients with poor cooperation since they accelerate the restorative procedure. Thus, bulk‐fill composites are particularly appealing for use in pediatric restorative procedures (Moorthy et al., 2012).

Bulk‐fill composites are available in low viscosity (flow) and high viscosity (restorative) types, and can be injected into the cavity and easily applied. The newest generation of bulk‐fill composites can also be used for posterior restorations in depths over 4 mm due to improved mechanical properties. In new‐generation bulk‐fill composites, the advanced monomer technology decreases the polymerization shrinkage and has advantages such as lower cuspal flexure in standard class II cavities, and optimal adaptation to the cavity walls (Dahl, 2016). Adverse complications such as postoperative tooth hypersensitivity, microleakage and debonding also have a lower frequency in use of new‐generation bulk‐fill composites (El‐Damanhoury & Platt, 2014). The bulk application technique has fewer clinical steps and accelerates the procedure as such (Didem et al., 2014). However, some studies reported that use of new‐generation bulk‐fill composites had no significant effect on cervical microleakage (Gallo et al., 2000; Moorthy et al., 2012).

The main advantages of bulk‐fill composite resins are their increased depth of cure probably due to their high translucency, and low polymerization stress (Ilie et al., 2014). SonicFill is a bulk‐fill composite which is polymerized in 4‐mm‐thick increments, and possesses the properties of both flowable and packable composite resins at the same time. SonicFill has its own specific handpiece, that decreases the viscosity of composite by sonic energy activation, and aids in fast application of composite and its optimal adaptation to cavity walls. When the sonic energy is discontinued, the composite turns into viscous state, which is more suitable for shaping and carving (Leprince et al., 2014).

Since the introduction of bulk‐fill composite resins, their properties such as bond strength, cuspal flexure, degree of conversion, depth of cure, gap formation, mechanical properties, microleakage, and polymerization shrinkage have been the topic of many studies. However, the majority of such investigations have been conducted on permanent teeth. Thus, this study aimed to assess the microleakage at the enamel and dentin margins of standard class II cavities restored with a bulk‐fill and a conventional composite resin.

## 2 MATERIALS AND METHODS

This in vitro, experimental study was conducted on 60 sound primary molars extracted within the past 3 months due to their physiologic exfoliation time or serial extraction for orthodontic treatments. The study protocol was approved by the ethics committee of Tehran University of Medical Sciences (code: IR.TUMS.DENTISTRY.REC.1397.181). The sample size was calculated to be 25 in each group according to a study by Mosharrafian et al. (Mosharrafian et al., 2017) considering *α* = .05, *β* = .2, standard deviation of 2.4 and 343 µm for the two groups, and difference in microleakage values of 333 µm between the two groups using two‐sample independent *t*‐test.

The collected teeth were stored in saline. Before the onset of the experiment, the teeth were immersed in 0.5% chloramine T solution at 4°C for 1 week. Standard class II cavities were created in the mesial and distal surfaces such that they had 2 mm buccolingual width, and 1.5 mm isthmus width, and their cervical margin was 1 mm below the cementoenamel junction (CEJ). The teeth were randomly divided into two groups (*n* = 30). The cavities were restored with SonicFill bulk‐fill composite (Kerr) in group 1, and Filtek Z250 conventional composite (3 M ESPE) in group 2. Single Bond 2 adhesive was used in both groups (Table 1). The conventional composite was applied incrementally by manual instruments while the bulk‐fill composite was applied as bulk.

**Table 1:** Characteristics of composite resins and adhesive used in this study.

Material	Composition	Manufacturer
Filtek Z250 (A2, N482264)	Bis‐GMA, Bis‐EMA, TEGDMA, UDMA Zirconia, silica (82% wt, 60% vol)	3M, ESPE
SonicFill (A2, 3026722)	Bis‐GMA, TEGDMA, EBPDMA silica, glass, oxide (83.5% wt, 42.5% vol)	Kerr
Single Bond 2 total‐etch self‐priming	Dimethacrylate resins, HEMA, VitrebondTM copolymer, filler, ethanol, water, initiation	3M, ESPE

In group 1, the cavities were rinsed and dried, enamel was etched for 20 s, and dentin was etched for 15 s, followed by 10 s of rinsing and drying. Two layers of Single Bond 2 were applied, air‐thinned for 5 s at 10 mm distance, and cured for 20 s. Bulk‐fill composite was applied as mass, cured for 40 s using a LED curing unit (Woodpecker) with a light intensity of 800–1000 mW/cm^2^, and the surface of specimens was polished.

In group 2, the same steps were performed for adhesive application as explained for group 1. Next, conventional composite resin was applied incrementally in two steps, and after curing for 20 s, the surface of specimens was polished.

**Figure 1:**
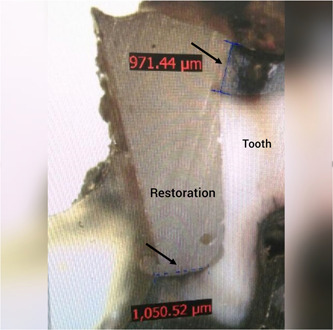
Measuring the dye penetration depth (indicative of microleakage) in a tooth specimen under a stereomicroscope at ×10 magnification.

The surface of the teeth was then coated with two layers of nail varnish except for a 1 mm margin around the restorations. Tooth apices were also sealed with wax. The teeth then underwent 1500 thermal cycles between 5°C and 55°C with a dwell time of 30 s and a transfer time of 15 s. Next, the teeth were immersed in water at 37°C for 24 h. They were then immersed in 1 M silver nitrate solution in the dark for 6 h, and after rinsing with water, they were immersed in developing solution for 12 h, and exposed to fluorescent light. After drying, the teeth were mesiodistally sectioned by a high‐speed diamond cutter (Mecatome R210A) under water coolant. The sections were inspected under a stereomicroscope (EZ4D; Leica, Olympus) at ×10 magnification and digital photographs were taken from the specimens to measure the dye penetration depth. The dye penetration depth was measured and recorded by an examiner blinded to the group allocation of the teeth (Figure 1). The microleakage data were analyzed by SPSS version 25. The measures of central dispersion were reported for microleakage at the enamel and dentin margins in the two composite groups. Since the Kolmogorov–Smirnov test showed non‐normal distribution of data (*p* < .05), comparisons were made using the nonparametric Mann–Whitney U test. Level of significance was set at 0.05.

## 3 RESULTS

Table 2 presents the measures of central dispersion for dye penetration depth (indicative of microleakage) in the two composite groups. According to the Mann–Whitney U test, microleakage at the enamel margins of bulk‐fill composite group was slightly greater than that in the conventional composite group but this difference was not significant (*p* = .76). Conversely, microleakage at the dentin margins of the conventional composite group was slightly, but not significantly, higher than that in bulk‐fill composite group (*p* = .16). However, in both Filtek Z250 conventional composite and SonicFill bulk‐fill composite groups, microleakage at the dentin margins was significantly greater than that at the enamel margins (*p* = .0001 in both).

**Table 2:** Measures of central dispersion for the dye penetration depth (indicative of microleakage) at the enamel and dentin margins in the bulk‐fill and conventional composite groups.

Margin	Composite	Mean	Standard deviation	Minimum	Maximum	*p* Value	
Enamel	Filtek Z250 conventional	298.53	491.14	0	2025.73	0.76	0001.0
Enamel	SonicFill bulk‐fill	323.27	466.99	0	1655.75		
Dentin	Filtek Z250 conventional	1045.63	435.29	0	1744.36	0.16	
Dentin	SonicFill bulk‐fill	910.61	399.37	0	1522.73		

Table 3 presents the frequency percentage of dye penetration depth qualitatively in the two composite groups.

**Table 3:** Frequency percentage of dye penetration depth qualitatively in the two composite groups.

	Filtek Z250 dentin (%)	SonicFill dentin (%)	Filtek Z250 enamel (%)	SonicFill enamel (%)
Outer third	2.15	5.19	3.78	6.66
Middle third	9.23	26.1	8.10	9.23
Inner third	8.60	3.54	8.10	8.10

## 4 DISCUSSION

This study assessed the enamel and dentin microleakage of class II cavities of primary molars restored with a bulk‐fill and a conventional composite. The results showed the occurrence of microleakage at the enamel and dentin margins in both groups. However, no significant difference existed in microleakage between the two composite groups at the enamel or dentin margins. In other words, both conventional and bulk‐fill composites evaluated in this study had comparable performance regarding microleakage. This finding may be attributed to the fact that SonicFill is converted to a flowable composite for superior marginal integrity and adaptation, and minimizes microleakage as such. Also, the Filtek Z250 matrix is composed of siloxane and oxirane molecules, which cause <1% volume polymerization shrinkage, and subsequently decrease microleakage (Weinmann et al., 2005).

Sooraparaju et al. (Sooraparaju et al., 2014). evaluated the microleakage in class V cavities restored with Tetric‐N‐Ceram nano‐hybrid, Tetric‐N‐Flow flowable, and G‐aenial Universal Flow injectable composite resins, and found no significant difference among them in microleakage at the occlusal margins. However, injectable composite showed lower degree of microleakage at the gingival margins. They found no significant difference in microleakage between the conventional nano‐hybrid and flowable composite, which was somehow in line with the present results, although they did not use bulk‐fill composite resins. AlSagob et al. (AlSagob et al., 2018). evaluated the marginal microleakage of Filtek Supreme conventional composite in 2 and 4 mm increments, Filtek Supreme flowable, and Surefil SDR Flow in class II cavities. They showed that the microleakage of bulk‐fill composites was comparable to that of conventional composites, which was in agreement with the present results, although they used a different type of bulk‐fill composite. Mosharrafian et al (Mosharrafian et al., 2017). evaluated the microleakage of SonicFill bulk‐fill and Z250 conventional composite resins in class II cavities of primary posterior teeth. Similar to the present study, they used Single Bond 2 adhesive. They found no significant difference in microleakage between the bulk‐fill and conventional composite resins, which was in agreement with the present observations. Similar methodology may explain the similarity in the results of the two studies.

However, Zhu and Zhu (Zhu & Zhu, 2017) assessed the occlusal and cervical margin microleakage of class V cavities restored with Tetric‐N‐Ceram bulk‐fill composite, Tetric‐N Flow, and N‐Ceram nano‐composite. They demonstrated that Tetric bulk‐fill composite had significantly lower microleakage at the cervical margin compared with other groups but no significant difference was noted among the three groups in microleakage at the occlusal margins. Their results were partly in line with the present findings, and the differences may be attributed to different types of cavities (class V in their study vs. class II in the present study), different types of teeth (permanent teeth in their study vs. primary teeth in the present study), different types of composite resins (Tetric‐N‐Ceram in their study vs. SonicFill in the present study), and different types of dye (methylene blue in their study vs. silver nitrate in the present study). Patel et al. (Patel et al., 2016). evaluated the microleakage in deep class II cavities restored with bulk‐fill composites and showed that bulk‐fill composites provided a strong bond at the cementoenamel junction, and therefore, can be used in deep class II cavities in 4‐mm‐thick increments. Webber et al. (Webber et al., 2014). concluded that SureFill bulk‐fill composite had comparable performance to conventional composite regarding microleakage in class II cavities. Eunice et al. (Eunice et al., 2012). assessed the enamel margin microleakage of SonicFill in class V restorations and found no significant difference in microleakage between SonicFill and Filtek Supreme XTE. Their results were in accordance with the present findings, although they used 99mTc‐pertechnetate solution for measurement of microleakage. They showed that application of SonicFill was less time consuming, and it had easier clinical application (Eunice et al., 2012). Moorthy et al. (Moorthy et al., 2012). assessed cuspal flexure and cervical microleakage of class II cavities restored with Grandio SO dimethacrylate and SDR and x‐tra base bulk‐fill flowable composites and showed that SDR and x‐tra base bulk‐fill flowable composites had no significant superiority to the conventional composite regarding microleakage. Their results were similar to the present findings, despite evaluation of different brands of composite resins. Rengo et al. (Rengo et al., 2015). compared bulk‐fill and conventional composite resins using 50% silver nitrate and micro‐CT and digital microscopy, and demonstrated their comparable performance with no significant difference regarding microleakage. Despite some methodological differences between the two studies in terms of type of teeth, evaluation of supragingival versus subgingival margins, and type of adhesives, their results were in agreement with the present results.

Optimal results regarding the microleakage of SonicFill bulk‐fill composite are probably due to the fact that this composite resin is converted into a flowable state after application for better adaptation and marginal integrity, and decreases the microleakage as such. It benefits from the easy application potential of flowable composites combined with optimal properties of packable composites; thus, there would be no need to use another composite on top of it. SonicFill is among the best bulk‐fill composite resins available in the market. Also, increased translucency of bulk‐fill composite resins improves their curing depth and leads to better light penetration into deeper layers, resulting in greater depth of cure (Ilie et al., 2013). Furthermore, SonicFill is activated by sonic energy, and can be applied in one layer with 5 mm thickness without increased translucency, which guarantees optimal esthetics (Jackson, 2012). Its modifiers decrease its viscosity with sonic energy by 87%, and increase its flow for better marginal adaptation with the cavity walls, and then it can be shaped following discontinuation of sonic energy (Jackon, 2011). Use of Single Bond 2 adhesive in the present study is another possible reason for lack of a significant difference in microleakage between the two composite groups (Campos et al., 2014).

The majority of available studies on bulk‐fill composites have been conducted on permanent teeth. Güngör et al (Güngör et al., 2014). measured the microleakage in class II cavities in permanent and primary teeth restored with a conventional composite and found no significant difference in microleakage at the occlusal margins. However, microleakage was significantly greater in the gingival margin of primary teeth than permanent teeth, which may be due to different enamel structure and thickness in primary teeth. In general, primary enamel has lower calcium and phosphorous content than permanent enamel, is thinner, and has higher density of rods (De Menezes Oliveira et al., 2010). Primary dentin has higher number of dentinal tubules with larger diameters, compared with permanent dentin, and thus, the available substrate for bonding is smaller in primary teeth (Sardella et al., 2005). All these factors can explain greater microleakage in primary teeth.

In the present study, microleakage at the dentin margins was significantly greater than that at the enamel margins in both groups. Also, dye penetration to the inner third (close to pulp chamber) had the highest frequency in the cervical margin of both groups, indicating high depth of microleakage at the dentin margins. Since the cavity margins were below the CEJ in the present study, such a high degree of microleakage may be justified. Dye penetration depth was lower in the study by Mosharrafian et al. (Mosharrafian et al., 2017) since the cavity margins were above the CEJ.

This study had some limitations. In vitro design was the main limitation of this study. The oral environment cannot be well simulated in vitro. Thus, generalization of in vitro results to the clinical setting must be done with caution. Also, it should be noted that leakage of fluids and dyes does not necessary mean leakage and proliferation of bacteria. Also, there is a possibility that the chemical composition of restorative materials and release of metal ions and fluoride from them decrease the occurrence of microleakage. Thus, long‐term clinical trials are required to make a final judgment regarding the performance of different restorative materials. Also, electron microscopy can be used in future in vitro studies for more precise assessment of microleakage. Microleakage of different types of conventional and bulk‐fill composite resins should be investigated in future studies as well.

## 5 CONCLUSION

Considering absence of a significant difference in microleakage, SonicFill bulk‐fill composite can be used as an alternative to Filtek Z250 conventional composite for restoration of primary molars to benefit from its advantages such as simpler and faster application.

## AUTHOR CONTRIBUTIONS

**Shahram Mosharrafian**: Conceptualization; data and formal analysis; investigation; methodology; project administration; supervision; validation; visualization; writing—original draft; writing—review and editing. **Niloofar Farahmand**: Conceptualization; investigation; methodology; validation; visualization; writing—original draft; writing—review and editing. **Kiana Poorzandpoush**: Conceptualization; data and formal analysis; investigation; methodology; project administration; supervision; validation; visualization; writing—original draft; writing—review and editing. **Zohre Sadat Hosseinipour**: Conceptualization; Investigation; methodology; validation; visualization; writing—original draft. **Mehdi Kahforushan**: Conceptualization; data and formal analysis; investigation; methodology; project administration and visualization.

## CONFLICT OF INTEREST STATEMENT

The authors declare no conflict of interest.

## Data Availability

The data used to support the findings of current study are available upon request from the corresponding author.
